# Fecal carriage of extended-spectrum β-lactamase-producing enterobacterales from hospitals and community settings in Gaza Strip, Palestine

**DOI:** 10.1186/s12866-023-03102-6

**Published:** 2023-11-30

**Authors:** Nabil Abdullah El Aila, Nahed Ali Al Laham, Basim Mohammed Ayesh, Thierry Naas

**Affiliations:** 1 Department of Medical Laboratory Sciences, Faculty of Applied Sciences, Al-Aqsa University Gaza, Gaza, Palestine; 2 Department of Laboratory Medicine, Faculty of Applied Medical Sciences, Al Azhar University-Gaza, Gaza, Palestine; 3 Bacteriology-Hygiene unit, Hôpital Bicêtre, AP-HP Paris-Saclay, Le Kremlin-Bicêtre, France; 4 LabEx LERMIT, Faculty of Medicine, Team ReSIST, UMR1184, INSERM, Université Paris-Saclay, Le Kremlin-Bicêtre, CEA France; 5 French National Reference Center for Antimicrobial resistances, Hôpital Bicêtre, AP- HP Paris-Saclay, Le Kremlin-Bicêtre, France

**Keywords:** ESBL, Fecal carriage, Hospitals, Community, Enterobacterales, Gaza Strip

## Abstract

**Background:**

The fecal carriage of extended-spectrum β-lactamase-producing Enterobacterales (ESBL-PE) is a major driver of the global spread of these antibiotic resistance determinants. Here we determined the rate of fecal ESBL-PE carriage in pediatric hospitals and community-serving healthcare centers serving adults and children in the Gaza Strip, Palestine.

**Methods:**

A total of 373 fecal and rectal samples were collected from different hospitals and clinics in Gaza. The antibiotic susceptibility was determined using the disk diffusion method and interpreted according to CLSI guidelines. The bacterial isolates were tested for ESBL production using phenotypic methods (double disk synergy test and growth on selective chromogenic media). *Bla*_CTX−M_, *bla*_SHV_, and *bla*_TEM_ genes were sought by PCR.

**Results:**

Out of the 373 isolates tested, 138 (37%) were considered ESBL positive as revealed by phenotypic tests. The prevalence of ESBLs among hospitalized patients was 39.1% (hospital setting) whereas, among outpatients attending community healthcare centers, it was 35.1% (community setting). ESBL production among *Escherichia coli*, *Klebsiella pneumoniae*, *Citrobacter freundii*, *Proteus mirabilis*, and *Klebsiella aerogenes* isolates was 52.8%, 39.1%, 26.7%, 2.8%, and 2.1% respectively. Meropenem and amikacin were the most effective antibiotics against ESBL producers (68.9% and 73.6% susceptibility, respectively), while only 15.2%, 22.5%, and 24.6% remained susceptible to ceftazidime, cefotaxime, and ceftriaxone, respectively. Out of 138 phenotypically ESBL-positive isolates, 98 randomly chosen were screened for *bla*_CTX−M_, *bla*_TEM_, and *bla*_SHV_ genes. The prevalence rate of *bla*_CTX−M_ was 45.9%, while *bla*_TEM_ and *bla*_SHV_ genes were detected in 16.8% and 5.2% of CTX-M-negative isolates (corresponding mostly for *K. pneumoniae* isolates in the case of SHV-PCR), respectively.

**Conclusions:**

The study revealed an alarmingly high prevalence of fecal carriage of ESBL-producing Enterobacterales among hospitalized children but also in the community of the Gaza Strip. In addition, 30% of ESBL-producers were already resistant to carbapenems, the treatment of choice of infections with ESBL-producers.

## Introduction

Extended-Spectrum β-Lactamase -Producing Enterobacterales (ESBL-PE) emerged as a serious threat worldwide in hospitals and community settings and infections with these bacteria are usually challenging as therapeutic options are drastically reduced [1–3]. Bacteria colonizing the human intestine are an important reservoir of resistance genes that contributes to the spread of ESBL-PE but also of ESBL-encoding genes in communities and in hospitals [4, 5].

ESBL are enzymes primarily produced by a variety of Gram-negative bacilli, in particular *Klebsiella pneumoniae* and *Escherichia coli* [6, 7]. They are also produced by non-fermentative Gram-negative organisms, such as *Acinetobacter baumannii* and *Pseudomonas aeruginosa* [8]. ESBL are β-lactamases capable of conferring bacterial resistance to penicillins, first, second, and third-generation cephalosporins (3GC), and aztreonam (but not cephamycins or carbapenems) through ß-lactam hydrolysis, and they are inhibited by clavulanic acid and tazobactam [7]. These enzymes are often encoded by genes located on large plasmids, and these also carry genes for resistance to other antimicrobial agents such as aminoglycosides, trimethoprim, sulphonamides, tetracyclines, and chloramphenicol [9]. Initially, ESBLs were derived from TEM and SHV-type β-lactamases, which were mainly produced by *K. pneumoniae*, a major cause of hospital-acquired infections. CTX-M-type ESBLs emerged in early 2000 and have become within 10 years the main ESBLs produced in hospitals and in the community, especially for *E. coli* [10, 11].

The horizontal transfer of resistance genes carried on plasmids is thought to be one of the primary causes of the dissemination of MDR isolates [12]. The human intestinal tract provides an important reservoir for ESBL-producing bacteria and colonized persons are at risk for developing infections due to ESBL producers and at risk for their dissemination [13–15]. In hospital settings, patient-to-patient transmission of these bacteria may occur [13, 16, 17]. The widespread use of 3GC and fluoroquinolones has been pointed out as a major cause for the selection of ESBL producers [18]. In addition, bacterial infections due to ESBL-PE have been associated with improper antibiotic use, increased hospital costs, length of stay, and patient mortality [19].

The highest ESBL-PE carriage prevalence has been described in Asia, whereas prevalence rates are lower in Europe and North America [20–22]. Before 2008, the prevalence of community-acquired ESBL-PE in Thailand was low (less than 10%) but has rapidly increased to more than 60% in recent years, posing a significant challenge for antibiotic therapy to combat both nosocomial and also community-acquired infections [23, 24].

Several studies have described the fecal carriage of ESBL-PE in the community and hospital settings [25–28]. Great variations were found in the ESBL-PE carriage rates in the Eastern Mediterranean, with rates ranging from 28% in Tunisia to 43% in Lebanon, to 70% in Egypt [29–31].

In Gaza Strip, Palestine, previous studies have shown high rates of ESBL-PE responsible of infections in two hospitals, with 22% in Naser and 37.5% in Al Shifa hospitals [32, 33].

However, the fecal carriage of ESBL-PE has never been addressed previously in Palestine in community settings. The Gaza Strip (31°250 N, 34° 200 E) is a narrow territory (41 km long and 6–12 km wide) along the eastern Mediterranean coast, with tightly controlled borders abutting Israel and Egypt’s Sinai Peninsula. It is regarded as one of the world’s most densely populated areas, with a population of approximately 2.1 million inhabitants (37.5% of the total estimated Palestinian population) and a population density of 4,073/km^2^, nearly ten-fold more than in the West Bank (433/km^2^) [34].

The present study aimed to investigate the prevalence of fecal carriage of ESBL-PEs in hospitalized paediatric patients and outpatients attending community healthcare centers in the Gaza Strip.

## Materials and methods

### Study design

The study was set out as a cross-sectional study in three pediatric hospitals and three Primary Health Care (PHC) clinics located in Gaza city. Among the 373 samples analyzed, 200 were fecal samples (53.6%) from outpatients attending community healthcare centers including Al-Remal, Al-Sourani, and Sabha Harazin clinics and 173 were rectal swab samples (46.4%) from hospitalized infants in the nursery departments of Al-Naser, Al-Rantisi and Al-Shifa hospitals. The average age of those children was from 2 days to 2 years. However, most of them were below 1 year of age.

The samples were collected from March to September 2019. The study was approved by the department of human resources and development of the Ministry of Health – Gaza and the local Helsinki committee under the study approval number PHRC/HC/226/17.

### Culture of isolates

Fecal and rectal samples were transported in Amies transport medium to the laboratory of microbiology of the Al Aqsa University – Gaza, where they were spread and incubated at 37 °C overnight on MacConkey agar plates (HiMedia, Mumbai, India). Bacterial isolates were identified upon cultural characteristics and relevant biochemical galleries (API, bioMérieux, Marcy-l-Etoile, France).

### Antimicrobial susceptibility testing

Antimicrobial susceptibility testing of clinical isolates was performed by Kirby Bauer disc diffusion method as recommended by CLSI guidelines [35] using Mueller-Hinton agar. Before inoculation, the swab stick was dipped into a bacterial suspension having visually equivalent turbidity to a 0.5 McFarland standard. The antibiotics used in this study were those currently available in Palestinian hospitals to treat Gram-negative infections: Amoxicillin-clavulanic acid (20/10 µg), Cefotaxime (30 µg), Ceftriaxone (30 µg), Ceftazidime (30 µg), Gentamicin (10 µg), Amikacin (30 µg), Ciprofloxacin (5 µg), Imipenem (10 µg), Amoxicillin (30 µg), Cephalexin (30 µg), Cefuroxime (30 µg), Co-Trimoxazole (25 µg), Meropenem (10 µg) and Doxycycline (30 µg) (HiMedia, Mumbai, India). The zone of inhibition was interpreted for each antimicrobial agent, reporting the organism as resistant, intermediate, or susceptible according to CLSI [35].

### Phenotypic detection of ESBLs using double disk synergy test

To infer the presence of an ESBL, synergy image between ceftriaxone (30 µg), cefotaxime (30 µg), ceftazidime (30 µg), and amoxycillin/clavulanic acid (20/10 µg) containing discs were monitored. The three 3GC containing discs were placed at 20 mm (edge to edge) from the Amoxycillin/Clavulanic acid disc that was placed in the middle of the plate. After 24-h incubation, if an enhanced zone of inhibition between either of the Cephalosporin antibiotics and the Amoxycillin/Clavulanic acid disc occurred, the test was considered positive for the production of ESBL. *K. pneumoniae* ATCC 700,603 and *E. coli* ATCC 25,922 were used as positive and negative controls, respectively [36]. We have done the calculation of MDR by counting the isolates which are resistant to three different families.

### Phenotypic detection of ESBLs using CHROMagar ESBL

Each bacterial isolate was cultured on CHROMagar ESBL agar and was incubated aerobically at 37 °C for 18 to 24 h. Colonies of ESBL producers develop species-specific colors (dark pink to reddish for *E. coli*; metallic blue for *Klebsiella* spp.; and a brown halo for *P. mirabilis*). Non-Enterobacterales and non-ESBL-producers grew with colorless colonies or not at all on CHROMagar ESBL agar, respectively [37].

### DNA extraction

DNA was extracted from cultured bacteria by a rapid alkaline lysis as previously described [38]. Briefly, one bacterial colony was suspended in 20 µL of lysis buffer (0.25% sodium dodecyl sulfate, 0.05 N NaOH) and heated at 95 °C for 15 min. The cell lysate was diluted with 180 µL of distilled water. The cell debris was pelted by centrifugation at 16 000 x g for 5 min. and the supernatants were used for polymerase chain reaction (PCR) or frozen at -20 °C until further use.

### Detection of ***bla***_CTX−M_, ***bla***_TEM,_ and ***bla***_SHV_ genes by PCR

The sequences of the primers used for the detection of the SHV gene were 5’GCC CGG GTT ATT CTT ATT TGT CGC-3’as a forward primer and 5’- TCT TTC CGA TGC CGC CGC CAG TCA-3’ as a reverse primer. The two primers amplify a 1016 bp fragment [39]. For detection of the CTX-M gene, the sequences of the primers used were 5’-ACC GCG ATA TCG TTG GT-3’as a forward primer and 5’-CGC TTT GCG ATG TGC AG-3’ as a reverse primer. The two primers produce a 550 bp amplicon [40]. For detection of the TEM gene, the sequences of the primers used were 5’- ATG AGT ATT CAA CAT TTC CG-3’as a forward primer and 5’- CCA ATG CTT AAT CAG TGA GG-3’ as a reverse primer. The two primers produce an 858 bp amplicon [41].

The reactions were performed in 25 µl final volumes in the presence of 1 µM of each primer, 2 µl of DNA, and 1X of the GoTaq Green MMX (Promega, Madison, USA). The thermal cycling program for detection of the SHV, CTX-M, and TEM genes was as follows: one cycle of initial denaturation at 95 °C for 5 min; followed by 34 cycles of denaturation at 95 °C for 30 s, annealing for 30 s at the proper temperature (54 °C for SHV, 55 °C for CTX-M and 68 °C for TEM), and extension at 72 °C for 1 min. A final extension step was performed at 72 °C for 5 min. The amplified products were resolved on a 2% agarose gel. The fragments were stained with ethidium bromide and visualized and photographed using a gel documentation system. A 100 bp ladder was run as a molecular weight marker (Bioline, Meridian Bioscience, Memphis, TN, USA). A positive control and blank negative control were tested in each run to exclude contamination.

### Statistical analysis

The results were tabulated and analyzed using version 20 of the Statistical Package for the Social Sciences (SPSS). Frequencies, cross-tabulation, and appropriate statistical tests such as Chi-square test and Fisher exact test were performed. A *P-*value of less than 0.05 was considered significant.

## Results

### Phenotypic detection

The number of samples that were collected from the hospitals Al-Shifa, Al-Naser, and Al-Rantisi was: 65 (38%), 65 (38%), and 41 (23.9%), respectively. The number of samples from the clinics Al-Remal, Sabha Harazin, and Al-Sourani was 97 (48%), 50 (24.7%), and 55 (27.3%), respectively (Table 1). Thus, a total of 373 samples were plated on MacConkey agar plates. A single colony from every bacterial isolate has been retained for any further investigation, encompassing bacterial identification, susceptibility testing, and ESBL production testing. Hundred and thirty-eight (37%) were considered positive phenotypically for ESBL production as revealed by the synergy testing and 235 (63%) were considered non-ESBLs based on absence of synergy image. In addition, these isolates did not grow on ChromAgar ESBL media, a media used to screen for ESBL-PE. In comparison between hospitals and community-serving healthcare centers, the prevalence of ESBL was similar, 39.1% and 35.1%, respectively.

The ESBL production rates at AL-Rantisi Hospital, AL-Nasser Hospital, and AL-Shifa Hospital were 24.3%, 36.9%, and 50.7%, respectively. In contrast, the prevalence of ESBL at Al-Remal Clinic, Sabha Harazin Clinic, and AL-Sourani Clinic was 42.2%, 34.5%, and 22%, respectively (Table 1).

Regarding the bacterial isolates, *E. coli* accounted for 57.9% (n = 216), with 63 from hospitalized patients and 153 from community outpatients. *K. pneumoniae* constituted 34.1% (n = 127), with 87 from hospitalized patients and 40 from community outpatients. Other isolates included *C. freundii* at 4% (n = 15), *K. aerogenes* at 2.6% (n = 10), and *P. mirabilis* at 1.3% (n = 5) (Table 1).

In hospitals, the predominant ESBL-producing species was *K. pneumoniae*, accounting for 54.5% (36 cases), followed by *E. coli* at 31.8% (21/66), *C. freundii* at 4.5% (3), *P. mirabilis* at 6% (4), and K. aerogens at 3% (2). Among the community patient clinic population, E. coli was the most common ESBL producer at 72.2% (52/72), followed by *K. pneumoniae* at 25% (18/ 72 cases), *C. freundii* at 1.4% (1), and *K. aerogenes* at 1.4% (1) (Table 1).

**Table 1:** Prevalence of ESBL-producing isolates among microbial species from Gaza hospitals and clinics

	AL-Rantisi hospital		AL-Nasser hospital		AL-Shifa hospital		Al-Remal clinic		Sabha Harazin clinic		AL-Sourani clinic		TOTAL	
	N	%	N	%	N	%	N	%	N	%	N	%	N	%
Number of isolates	41	10.9	65	17.4	65	17.4	97	26	55	14.7	50	13.4	373	100
ESBL-phenotype	10	24.3	24	36.9	33	50.7	41	42.2	19	34.5	11	22	138	37
*E. coli*	9 (28)	21.9	4 (13)	6.1	8 (22)	12.3	23 (60)	23.7	18 (52)	32.7	11 (41)	26.8	73 (216)	52.8
* K. pneumoniae*	0 (12)	0	15 (43)	23	21 (32)	32.3	17 (33)	17.5	1 (1)	1.8	0 (6)	0	54 (127)	39.1
* C. freundii*	0 (1)	0	2 (3)	3	1 (7)	1.5	1 (2)	2.4	0 (0)	0	0 (2)	0	4 (15)	26.7
*P. mirabilis*	1 (1)	2.4	1 (2)	1.5	2 (2)	3	0 (0)	0	0 (0)	0	0 (0)	0	4 (5)	2.8
* K. aerogenes*	0 (1)	0	1 (2)	1.5	1 (2)	1.5	1 (3)	1	0 (1)	0	0 (1)	0	3 (10)	2.1

( ): It is the total number of the isolates

The ESBL rate in hospitals varied between the different departments. For example, in the AL-Rantisi hospital, the prevalence rate of ESBL among the departments of dialysis, neurology, and hematology/oncology was 50%, 23.1%, and 10%, respectively (*P* value = 0.05). The ESBL rate in the nursery and pediatric ICU departments in the Al-Naser hospital was 55.2% and 44.5%, respectively (*P* value = 0.390), whereas the prevalence rate in these departments in the AL-Shifa hospital was 57.6% and 17.9%, respectively (*P* value = 0.002). The highest rate of ESBL production was found in the nursery department among the departments in both the Al-Shifa and Al-Naser hospitals (Table 2).

**Table 2:** Prevalence of ESBL-producing isolates among the different departments of the AL-Rantisi, AL-Naser, and AL-Shifa hospitals

Hospital	AL-Rantisi			AL-Naser		AL-Shifa	
Department	Hematology/ Oncology	Dialysis	Neurology	Pediatric ICU	Nursery	Nursery	Pediatric ICU
ESBL	2	5	3	12	21	19	5
	11.1%	50%	23.1%	44.4%	55.3%	57.6%	15.6%
*P*-value	0.002			0.390		0.002	

### Genotypic detection

Out of the 138 isolates with ESBL phenotype, 98 were randomly screened for *bla*_CTX−M_, *bla*_TEM,_ and *bla*_SHV_ genes. These bacteria were 31 *E. coli*, 56 *K. pneumonia*e, 4 *C. freundii*, 3 *P. mirabilis* and 4 *K. aerogenes*. Using PCR, 60 isolates (61.2%) were positive for at least one gene. The *bla*_CTX−M_ gene was detected in 13 (41.9%) *E. coli*, 27 (48.2%) *K. pneumoniae*, 2 (50%) *C. freundii*, 1(33.3%) *P. mirabilis* and 2 (50%) *K. aerogenes* isolates (Table 3), e.g. 45.9% of the phenotypically suspected ESBL-producing Enterobacterales.

The results of TEM and SHV PCR were considered only in the CTX-M-negative isolates. Out of the 54.1% CTX-M-negative isolates, which was composed of 18 *E. coli*, 29 *K. pneumonia*e, 2 *C. freundii*, 2 *P. mirabilis* and 2 (50%) *K. aerogenes*, the positivity of *bla*_TEM_ in these species was 27%, 24%, 50%, 100% and 50% respectively, and for *bla*_SHV_ it was 42.8% for *K. pneumoniae*, 50% for *C. freundii* and 100% for *K. aerogenes* isolates, respectively. For 38 isolates, no positive PCR was evidenced, suggesting that a novel and undetectable CTX-M, TEM, or SHV-variant be present, or simply an ESBL that was not screened for, such as GES, PER, BEL, and others.

**Table 3:** Prevalence of each gene among ESBL-PE

	Total of screened isolates	CTX-M pos.		TEM in CTX-M neg. PCR		SHV in CTX-M neg. PCR	
	N	N	%	N	%	N	%
*Escherichia coli*	31	13	41.9	5	16.1	0	0
*Klebsiella pneumoniae*	56	27	48.2	7	12.5	3	5.3
*Citrobacter freundii*	4	2	50	1	25	1	25
*Proteus mirabilis*	3	1	33.3	2	66.6	0	0
*Klebsiella aerogenes*	4	2	50	1	25	1	25
Total	98	45	45.9	16	16.3	5	5.1

### Antibiotic profile

Based on the overall characteristics of antibiotics, meropenem was found to be the most efficient on the 373 Enterobacterales, with a susceptibility rate of 74.5%, followed by amikacin with 74.3%. On the other hand, amoxicillin and amoxicillin-clavulanic acid were the least effective antibiotics, with a resistance rate of 72.9% and 68.4%, respectively.

Out of the 138 ESBL producers, amikacin and meropenem demonstrated the highest effectiveness against them, with susceptibility rates of 73.6% and 68.9%, respectively. On the other hand, ceftazidime, cefotaxime, and ceftriaxone had high resistance rates of 84.8%, 77.5%, and 75.4%, respectively. Additionally, the resistance rate for amoxicillin was also high, reaching up to 79.7%.

Among the 235 non-ESBL producers, a higher susceptibility rate was found for meropenem and amikacin, with 84.1% and 75.4%, respectively. However, the resistance rates for amoxicillin and amoxicillin-clavulanic acid still very high with 68.9% and 62.3% of resistant bacteria, respectively, as indicated in Table 4.

Among the ESBL producers, 47.1% (65/138) had resistance to eight antibiotics or more, while among the non-ESBL producers, the prevalence was lower at 26.4% (62/235). Additionally, the prevalence of multiple drug resistance (MDR) was higher among ESBL isolates, with 90.6% (125/138) compared to non-ESBL producers, where the prevalence was 72.8% (171/235).

**Table 4:** Comparison of susceptibility profiles between ESBL and non-ESBL producing isolates

Antibiotic	ESBL (138)						Non-ESBL (235)						*P-* value
	**S**		**I**		**R**		**S**		**I**		**R**		
	%	N	%	N	%	N	%	N	%	N	%	N	
Amoxicillin	**8.7**	12	11.6	16	79.7	110	**19.1**	45	11.9	28	68.9	162	0.022
Amoxicillin-clavulanic acid	**8**	11	29.7	24	71.9	86	**17.9**	42	10.2	41	62.3	169	< 0.001
Cefuroxime	**18.1**	25	26.8	37	55.1	76	**40.9**	96	23.4	55	35.7	84	< 0.001
Cephalexin	**31.2**	43	10.1	14	58.7	81	**43.8**	103	12.3	29	43.8	103	0.02
Cefotaxime	**22.5**	31	0	0	77.5	107	**60.4**	142	5.1	12	34.5	81	< 0.001
Ceftriaxone	**24.6**	34	0	0	75.4	104	**57.4**	135	6.4	15	36.2	85	< 0.001
Ceftazidime	**15.2**	21	0	0	84.8	117	**47.2**	111	12.8	30	40	94	< 0.001
Imipenem	**32.6**	45	40.6	56	26.8	37	**40.9**	96	36.1	85	23	54	0.272
Amikacin	**73.6**	173	1.3	3	25.1	59	**75.4**	104	0.7	1	23.9	33	0.576
Gentamicin	**38.4**	53	23.2	32	37.7	52	**48.5**	114	19.1	45	32.3	76	0.165
Co-trimoxazole	**17.4**	24	23.2	32	59.4	82	**79**	33.6	27	11.5	54.9	129	< 0.001
Ciprofloxacin	**39.9**	55	34.8	48	25.4	35	**46.8**	110	23	54	30.2	71	0.046
Doxycycline	**14.5**	20	30.4	42	55.1	76	**31.1**	73	9.4	22	59.6	140	< 0.001

## Discussion

Fecal carriage of ESBL-PE is one of the primary mechanisms for their global spread in hospital and community settings worldwide. Extensive use of beta-lactam antibiotics in hospitals and community has created a major problem leading to increased morbidity, mortality, and health care costs [42]. In this study, we estimated and characterized the prevalence of ESBL-producing Enterobacterales in the community and hospital settings in Gaza Strip. No previous studies have investigated the prevalence of fecal carriage of ESBL in both settings in Gaza Strip.

In the present study, the overall fecal carriage of ESBL-PE was 37%. Among the participants, pediatric hospitalized patients showed a prevalence of 39.1%, whereas children and adults attending community healthcare centers exhibited a slightly lower rate of 35.1%, while it was 39.1% in pediatric hospitalized patients and 35.1% in adults and children outpatients seen in community healthcare centers.

This ESBL‑PE fecal carriage rate is quite high when compared to those reported in the Czech Republic, 8.2% in hospitalized patients and 3.2% in community subjects [43]; in Spain, 5.5% in outpatients and 3.7% in healthy volunteers [44]; in the Netherlands, 10.1% in community patients [45]; 16.7% in outpatients attending community health centers in Malawi [46] and 17.1% among children under five years in Ethiopia [47].

ESBL-PE are widely acknowledged as significant pathogens acquired in hospitals among children and are frequently associated with outbreaks. Our findings are consistent with other researchs. For instance, Akenten et al. discovered that the rate of stool carriage of ESBL-EC and ESBL-KP was 40.9% in children under 5 years old in the hospital [48]. Our study recorded a higher prevalence compared to another investigation that reported an overall fecal carriage rate of ESBL-producing *E. coli* and *K. pneumoniae* of 17.1% in children under five attending outpatient departments [47]. Furthermore, another study reported an even higher faecal ESBL carriage rate of 54% among the 171 children they examined [49].

In our research, we observed that 35.1% of community patients carried ESBL in their fecal samples. Similar high rates of fecal carriage of ESBL-PE have been observed, in Turkey with 34.3% and 30% among community outpatients and individuals attending routine check-up clinics, respectively [50, 51], and in Nepal with 30.92% among participants of the Out Patient Department [5]. Furthermore, our findings showed a higher prevalence compared to Onduru et al., in Blantyre, Malawi who reported a 16.67% prevalence of community-acquired ESBL-PE [46]. Our results were higher when compared to France (6.0%), Switzerland (5.8%), Japan (6.4%), Tunisia (7.3%), Germany (6.4%), and Libya (13.4%) or Malawi (16.67%). On the other hand, it is lower than the rates reported in China (50%), Egypt (63%), and Thailand (65.7%) [23, 46, 52–56].

Our findings suggest that ESBL-PE are present in hospital settings but also in the community at similar rates. Several factors contribute to ESBL-PE colonization and infection, including poor drug quality or inadequate posology, irrational antibiotic use, unskilled practitioners, self-medication, unsanitary conditions that contribute to the spread of resistant bacteria, and insufficient surveillance programs [57, 58].

In our study, different rates of ESBL production were observed according to the species, with as expected *E. coli*, and *K. pneumoniae (*52.8%, 39.1%) being the top species. Interestingly, ESBL-producing *K. pneumoniae* were also observed in the community, suggesting community spread of these bacteria.

Among the 153 *E. coli* isolates obtained from community patients, 53 (34.6%) were found to be ESBL producers, a notably higher percentage compared to findings from other studies. In a study conducted by Bezabih et al. in 2021, they reported a global pooled prevalence of ESBL *E. coli* intestinal carriage in the community to be 16.5% [25]. Moreover, ESBL production among *K. pneumoniae* was higher (39.1%) in comparison with a study conducted in Turkey (8%), whereas ESBL production among *E. coli* in Turkey (92%) was higher in comparison with our findings (52.8%) [59]. In another study conducted in Saudi Arabia, ESBL production in community isolates of *E. coli* was higher (95.6%) in comparison with our results, while ESBL production among *Klebsiella* spp. was lower (4.4%) [60].

### ESBL genotype

Out of 98 isolates screened for *bla*_CTX-M_, *bla*_TEM_, and *bla*_SHV_ genes using PCR, 60 (61.2%) have at least one gene. CTX-M enzymes are the most common ESBL types in both hospital and community settings worldwide [7, 61]. In our study, the *bla*_CTX-M_ gene was more prevalent than *bla*_TEM_ and *bla*_SHV_. These findings are consistent with previous reports from other countries that found CTX-M to be more dominant than other types [54, 56, 62, 63].

It is worth noting that the CTX-M ß-lactamase genes are widely known to be carried on plasmids that are used as vehicles for horizontal movement of resistance genes, in addition to carrying on resistance to other drugs such as aminoglycosides and fluoroquinolones [30, 64, 65].

Co-existence of all three *bla* genes was observed in 12 (12.3%) isolates in this study, which was consistent with the study of Saho et al., who detected their existence in 10.0% of isolates but higher than the report from North India where they observed only in 6.5% of the tested isolates [66]. The presence of TEM, SHV, and CTX-M, along with impermeability, can result in carbapenem resistance [67], which is a major cause of concern in Gaza Strip. Indeed, in our study carbapenem resistance was very high 24.3%, which could be linked also to the presence of carbapenemases.

In this study, the *bla*_CTX-M_ gene was detected in 45.9% of the ESBL-PE, which is lower in comparison to that reported by Erdogan who detected *bla*_CTX-M_ in 87.5% of the ESBL-PE [50] and also reported in other several studies [51, 53]. This low percentage of *bla*_CTX-M_ gene may be due to regional variation, sample selection, timeframe, and antibiotic usage. In addition, it may be linked to the spread of a variant not detected using our primers or the spread of another ESBL such as PER, VEB, or GES. Further investigations are necessary to tackle this issue.

The prevalence rate of *bla*_TEM_ and *bla*_SHV_ genes were 16.8% and 5.2% in CTX-M-negative isolates, respectively. The presence of *bla*_TEM_ and *bla*_SHV_ genes in these isolates may be at the origin of the ESBL-phenotype, but further sequencing of the PCR product is necessary to conclude. One striking result is that only 42% of *K. pneumoniae*were SHV positive. Possible explanations could be the presence of SHV alleles not detected with our primers, or the presence of *K. pneumoniae* subspecies quasipneumoniae, that expresses LEN-type enzymes, or *K. variicola* that expresses OKP-type enzymes, that cannot be easily distinguished using biochemical tests from *K. pneumoniae*.

### Antibiotic profile

Antibiotic resistance is a major scientific concern, both in hospitals and in the community. Exposure to 3GC was associated with a significant risk of ESBL carriage in our study. In the current study, ESBL-PE isolates showed high resistance rates against ceftazidime, amoxicillin, and cefotaxime (84.8%, 79.7%, and 77.5%) respectively.

The rate of resistance against doxycycline and cefotaxime was 55.1%. Previous studies have shown that ESBL-PE have a high resistance rate to 1st & 2nd generation cephalosporin, which are always used to treat infections caused by gram-negative bacteria [68]. We found that amikacin and meropenem were the most effective antibiotics against ESBL-PE (73.6%, and 68.9%) respectively. Our results are in close agreement with studies done in Saudi Arabia (meropenem 88%, amikacin 91%) [60], in central India (meropenem 87.5%, amikacin 83.92%) [20], Jimma, Ethiopia (amikacin 83.7%) [69], Addis Ababa, Ethiopia (meropenem 96.7%, amikacin 82.1%) [70], and India (meropenem 94.0%, amikacin 82.6%) [71].

High rates of carbapenem resistance is particularly worrisome especially in bacteria that are already resistant to multiple other classes of antibiotics, making infections caused by these organisms extremely difficult to treat. The rampant use of carbapenems for treating ESBL‑PE may lead to selection of carbapenem resistance.

The prevalence of resistance to four and eight antibiotics in ESBL-PE was 81.8% and 47.1%, respectively. The prevalence of MDR phenotype among ESBL-PE was 90.6% (125/138) and 72.8% (71/235) among non-ESBL producers. This is comparable with a study that reported the prevalence of MDR among ESBL producers to be 96.3%, whereas only 30% of the non-ESBL-producers were MDR strains [70].

### Limitations of this study

PCR for ß-lactamase gene detection (CTX-M, SHV, and TEM) was performed only on a part of the isolates due to limited financial resources. In addition, PCR products could not be sequenced, and thus we could only speculate that a TEM/SHV ESBL was present in case of a positive synergy test and negative CTX-M PCR. Similarly, we were unable to perform PCR tests for the detection of other ESBL and/or carbapenemase genes, which could be also present as meropenem resistance was almost 31% among ESBL-PE.

## Conclusions

To the best of our knowledge, this is the first study on the intestinal carriage of ESBL-PE in hospitalized and community patients in Gaza Strip. This study showed high fecal colonization of ESBL-PE that is similar in hospitals and community settings in Gaza Strip. Antibiotic resistance and MDR among ESBL-PE are higher than for non-ESBL isolates. ESBL-PE isolates showed resistance to meropenem in 30% of the cases, which is alarming because this antibiotic is the drug of choice for the treatment of MDR GNB bacterial infections. Further studies are now necessary to identify the underlying resistance mechanisms to carbapenems.

With high rates of ESBL-producing isolates, irrational use of 3GC must be discouraged to reduce the selection pressure on these MDR bacteria and avoid treatment failures. Rational antibiotic prescription based on local guidelines to prevent the development of bacterial resistance is highly recommended. It is important to identify ESBL colonization in hospitalized patients to prevent further spread in hospital settings, but also to the community. The intestinal carriage of ESBL-PE poses a significant public health challenge, emphasizing the urgent need to improve sanitation and the implementation of antibiotic stewardship.

## Data Availability

All data generated or analyzed during this study were included in this article.
